# Diagnostic agreement of nutritional screening tools in obesity-related knee osteoarthritis

**DOI:** 10.3389/fnut.2026.1887413

**Published:** 2026-09-23

**Authors:** Zhiyuan Fan, Bohao Yin, Chenjun Liu, Qi Ai, Huibin Zhang, Yifei Wei, Yingzhe Jin

**Affiliations:** 1 Department of Orthopedic, The Affiliated Lihuili Hospital of Ningbo University, Ningbo, China; 2 Department of Orthopaedic Surgery, Shanghai Sixth People’s Hospital Affiliated to Shanghai Jiao Tong University School of Medicine, Shanghai, China; 3 Department of Orthopedic Trauma, Wangjing Hospital, China Academy of Chinese Medical Sciences, Beijing, China; 4 Second Department of Bone and Joint Disorders, Wangjing Hospital, China Academy of Chinese Medical Sciences, Beijing, China

**Keywords:** concealed malnutrition, diagnostic agreement, GLIM, inflammatory burden, knee osteoarthritis, nutritional screening, obesity, sarcopenic obesity

## Abstract

**Objective:**

To evaluate the diagnostic agreement, ability to identify concealed malnutrition, and clinical risk stratification value of different nutritional screening tools in patients with obesity-related knee osteoarthritis (KOA).

**Methods:**

Single-center, retrospective, cross-sectional diagnostic agreement study. Consecutive patients with obesity-related KOA hospitalized between January 2022 and January 2024 were enrolled. Nutritional risk was assessed using NRS-2002, MUST, MNA-SF, GNRI, and CONUT; malnutrition was confirmed by the GLIM criteria.

**Results:**

Among 520 patients, GLIM-defined malnutrition was present in 146 (28.1%). Detection rates ranged from 17.1% (MUST) to 35.2% (CONUT). Multi-tool agreement was moderate (Fleiss’ κ = 0.42). CONUT showed the highest agreement with GLIM (κ = 0.58), MUST the lowest (κ = 0.29). Overall diagnostic discordance rate was 37.9%. MUST had the highest missed diagnosis rate for GLIM-defined malnutrition (42.5%), CONUT the lowest (13.0%). Concealed malnutrition (defined as GLIM-defined malnutrition with BMI ≥ 28 kg/m^2^ but without significant recent involuntary weight loss) was identified in 121 patients, accounting for 82.9% of GLIM-defined malnourished patients. Low muscle mass, high CRP, and higher WOMAC score were independently associated with concealed malnutrition. Adding CRP and muscle mass improved the AUC from 0.73 to 0.86 (NRI = 0.31, IDI = 0.09); sensitivity analyses using a modified GLIM reference that excluded these components confirmed that the improvement was not solely driven by criterion overlap.

**Conclusion:**

In this cohort, concealed malnutrition was highly prevalent among patients with obesity-related KOA, accounting for 82.9% of GLIM-defined malnutrition cases. Traditional BMI-dependent screening tools may systematically underestimate nutritional risk in this population. A strategy integrating laboratory indices, inflammatory burden, and muscle mass appears to provide better diagnostic agreement and risk stratification in this setting; however, these findings are derived from a single-center retrospective study and require prospective external validation.

## Introduction

Knee osteoarthritis (KOA) is a leading cause of chronic disability worldwide, with its burden rising due to population aging, obesity, and metabolic diseases. In 2020, approximately 595 million people had osteoarthritis (7.6% of the global population), and KOA cases are projected to increase by 74.9% by 2050 (1). The global prevalence of KOA among people aged ≥ 40 years is about 22.9% (2), and in 2021 there were about 375 million prevalent cases, causing 12 million disability-adjusted life years (3). Obesity is a central driver of KOA, acting not only through mechanical load but also via adipose-mediated chronic low-grade inflammation, insulin resistance, and metabolic dysregulation (4). Recent reviews emphasize that obesity and metabolic syndrome promote osteoarthritis through systemic inflammation, adipokine abnormalities, and cartilage-synovium-bone imbalance, positioning obesity-related KOA as a complex interplay of mechanical and metabolic inflammation (5). Thus, relying solely on BMI may overlook underlying inflammation, muscle loss, and nutritional disorders (6).

Malnutrition has traditionally been associated with underweight individuals, but this view is challenged in obesity and chronic diseases. Obese patients can present with energy excess, insufficient protein reserves, chronic inflammation, muscle loss, and impaired immune nutrition—a phenotype termed concealed or obesity-associated malnutrition (7). In KOA, pain, reduced activity, and inflammatory burden further promote muscle loss, worsening joint instability and function (8). A 2024 review indicated that sarcopenia and obesity synergistically increase KOA risk (9), and a 2024 meta-analysis confirmed that low muscle mass and sarcopenic obesity are associated with higher KOA risk (10). A 2025 study found that about one-quarter of obese KOA patients had malnutrition, linked to abnormal metabolic markers, lower self-efficacy, and poorer physical function (11). These findings suggest that obesity-related KOA carries a compound risk of high BMI, chronic inflammation, muscle loss, and nutritional insufficiency, easily masked by traditional body-size assessment.

Current nutritional screening relies heavily on tools such as NRS-2002, MUST, and MNA-SF, which incorporate BMI, weight loss, reduced intake, and disease severity (12). In obese individuals, high BMI may blunt the signal of low BMI as a malnutrition indicator, and short-term weight loss may be misinterpreted as beneficial (13). Perioperative orthopedic studies report preoperative malnutrition rates of 8.5-50% in arthroplasty patients, associated with infection, prolonged stay, and poor recovery (14), yet no unified screening strategy exists, and results vary across tools (15). In elderly orthopedic and neurosurgical patients, tools like NRS-2002, MNA-SF, and GNRI show inconsistent risk identification (16), and diagnostic performance in chronic diseases depends on disease type, inflammatory status, and reference standard (17). Therefore, a systematic comparison of diagnostic agreement among different nutritional screening tools is clinically necessary in obesity-related KOA.

The GLIM criteria provide a structured consensus for malnutrition diagnosis, requiring a combination of phenotypic (weight loss, low BMI, low muscle mass) and aetiologic (reduced intake, inflammation) criteria (18). Unlike traditional BMI-based screening, GLIM incorporates low muscle mass and inflammatory burden, offering a rational foundation for identifying concealed malnutrition in obese individuals (19). The 2025 updated consensus confirms its validity while emphasizing the importance of assessing low muscle mass and inflammation in complex settings (20). However, real-world GLIM application still shows methodological heterogeneity (21). Meanwhile, laboratory-based indices such as GNRI and CONUT, integrating albumin, lymphocyte count, and cholesterol, may more sensitively reflect protein reserves and immune nutrition (22). In orthopedic populations, these indices have been used to predict outcomes, but their agreement with GLIM and ability to identify concealed malnutrition in obesity-related KOA have not been systematically validated (23, 24).

Notably, a very recent study by Vieira et al. reported a 26.1% prevalence of GLIM-defined malnutrition in a small cohort of 46 patients with obesity-related KOA and demonstrated its association with poorer self-efficacy and physical function (11). However, that study did not systematically compare different nutritional screening tools nor quantify their diagnostic agreement with GLIM criteria. More importantly, it remains unknown whether traditional BMI-dependent tools systematically underestimate nutritional risk in this population and whether incorporating inflammatory burden and muscle mass improves risk classification. Therefore, the present study extends the evidence by: (1) comparing the diagnostic agreement of five commonly used screening tools (NRS-2002, MUST, MNA-SF, GNRI, and CONUT) with the GLIM criteria in a large cohort of 520 patients; (2) quantifying missed diagnosis rates and discordance patterns; (3) evaluating the incremental value of adding CRP and muscle mass to traditional models; and (4) examining the associations of inflammatory burden, muscle mass, and functional impairment with concealed malnutrition and sarcopenic obesity. Unlike traditional tool comparisons, this study also analyses diagnostic discordance, missed diagnosis patterns, and reclassification improvement in the context of obesity, thereby clarifying the limitations of BMI-guided screening strategies in obesity-related KOA. By integrating nutritional screening, GLIM diagnosis, inflammatory markers, muscle mass, and clinical outcomes, we aim to establish a more accurate framework for identifying concealed malnutrition and to provide a clinical basis for perioperative risk assessment and individualized nutritional management.

## Results

### Patient disposition and baseline characteristics

During the study period, 648 hospitalized patients with obesity-related KOA were screened, and 520 were included after exclusions (Figure 1). The mean age was 66.8 ± 8.7 years, 68.5% were female, and mean BMI was 31.9 ± 3.2 kg/m^2^. According to GLIM criteria, 146 patients (28.1%) were diagnosed with malnutrition. Compared with non-malnourished patients, those with malnutrition were older, had lower BMI, and higher proportions of type 2 diabetes, hypertension, and chronic inflammation (all P < 0.05). They also had higher CRP levels, greater prevalence of low muscle mass and sarcopenic obesity, worse WOMAC scores, more advanced K-L grades, longer hospital stays, and higher perioperative complication rates (all *P* < 0.001) (Table 1). These findings indicate that nutritional abnormalities are common in obesity-related KOA and are closely associated with chronic inflammation, muscle loss, and disease severity.

**FIGURE 1:**
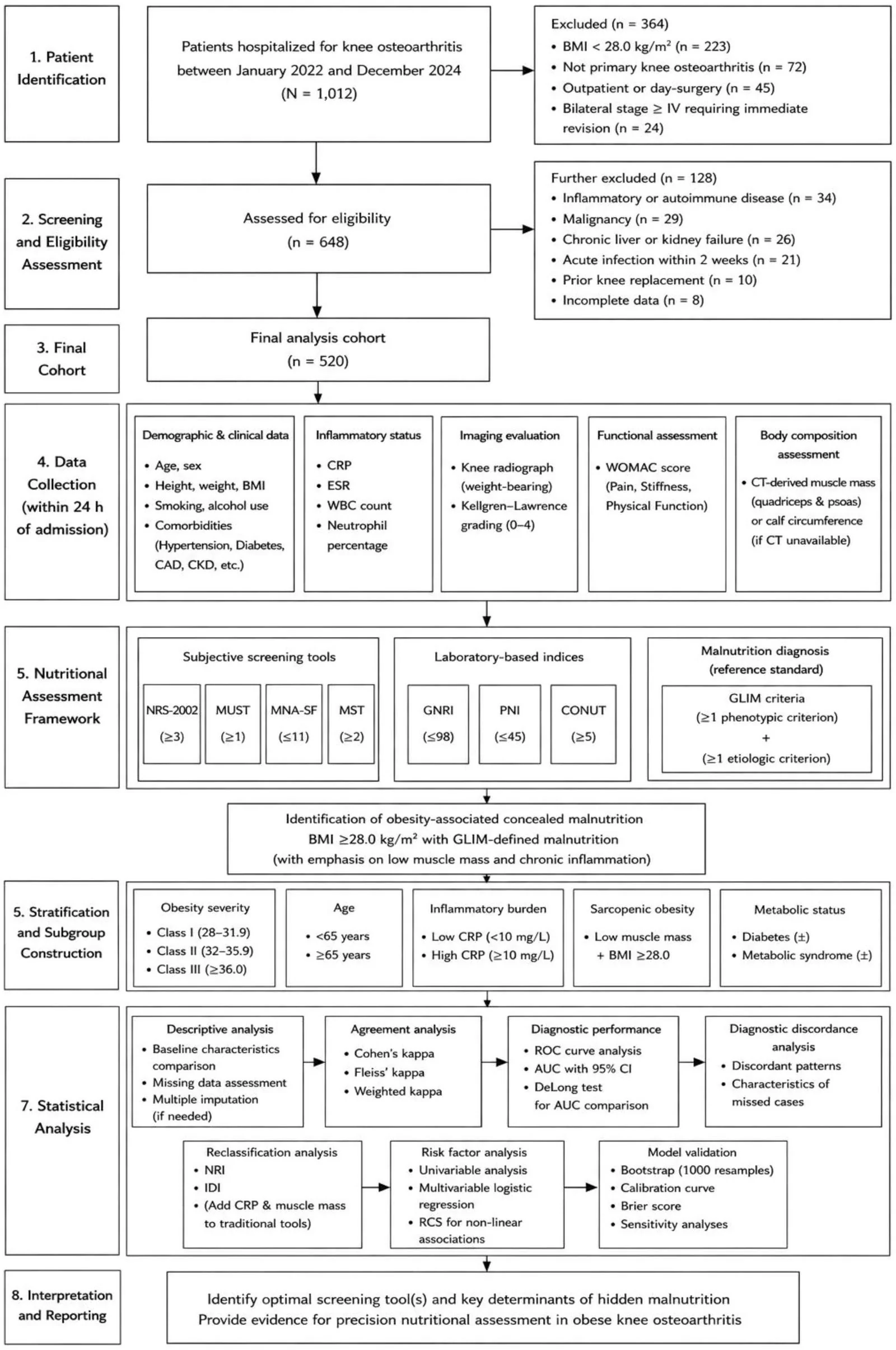
Flowchart of patient selection in obesity-related knee osteoarthritis. A total of 1,012 patients hospitalized for knee osteoarthritis between January 2022 and December 2024 were screened. After excluding 364 patients (BMI < 28.0 kg/m^2^, *n* = 223; not primary KOA, *n* = 72; outpatient or day-surgery, *n* = 45; bilateral stage ≥ IV requiring immediate revision, *n* = 24), 648 patients underwent eligibility assessment. A further 128 patients were excluded (inflammatory/autoimmune disease, *n* = 34; malignancy, *n* = 29; chronic liver/kidney failure, *n* = 26; acute infection within 2 weeks, *n* = 21; prior knee replacement, *n* = 10; incomplete data, *n* = 8), yielding a final cohort of 520 patients.

**TABLE 1:** Baseline characteristics of the study population according to GLIM-defined malnutrition status.

Variable	Total cohort (*n* = 520)	GLIM malnutrition (*n* = 146)	Non-malnutrition (*n* = 374)	*P*-value
Age, years	66.8 ± 8.7	69.4 ± 8.1	65.7 ± 8.6	< 0.001
Female sex, n (%)	356 (68.5)	108 (74.0)	248 (66.3)	0.089
BMI, kg/m^2^	31.9 ± 3.2	30.7 ± 2.9	32.4 ± 3.1	< 0.001
Class I obesity, n (%)	312 (60.0)	101 (69.2)	211 (56.4)	0.008
Class II obesity, n (%)	161 (31.0)	36 (24.7)	125 (33.4)	0.052
Severe obesity, n (%)	47 (9.0)	9 (6.2)	38 (10.2)	0.155
Smoking history, n (%)	138 (26.5)	49 (33.6)	89 (23.8)	0.027
Alcohol consumption, n (%)	102 (19.6)	34 (23.3)	68 (18.2)	0.188
Type 2 diabetes mellitus, n (%)	192 (36.9)	72 (49.3)	120 (32.1)	< 0.001
Hypertension, n (%)	338 (65.0)	108 (74.0)	230 (61.5)	0.007
Coronary artery disease, n (%)	104 (20.0)	39 (26.7)	65 (17.4)	0.018
Chronic kidney disease, n (%)	58 (11.2)	24 (16.4)	34 (9.1)	0.020
CRP, mg/L	7.8 (4.1–13.7)	12.6 (7.8–19.4)	6.1 (3.5–10.2)	< 0.001
High inflammatory burden (CRP ≥ 10 mg/L), n (%)	180 (34.6)	75 (51.4)	105 (28.1)	< 0.001
ESR, mm/h	28 (18–41)	37 (24–52)	24 (16–36)	< 0.001
Albumin, g/L	37.2 ± 4.6	33.9 ± 4.1	38.5 ± 3.9	< 0.001
Prealbumin, mg/L	214 ± 56	181 ± 48	227 ± 51	< 0.001
Total lymphocyte count, × 10^9^/L	1.72 ± 0.51	1.41 ± 0.42	1.84 ± 0.49	< 0.001
Sarcopenic obesity, n (%)	112 (21.5)	64 (43.8)	48 (12.8)	< 0.001
GLIM phenotypic criteria:				
–Involuntary weight loss, n (%)	25 (4.8)	25 (17.1)	0 (0)	< 0.001
–Low BMI ( < 18.5 kg/m^2^), n (%)	0 (0)	0 (0)	0 (0)	—
– Low muscle mass, n (%)	138 (26.5)	79 (54.1)	59 (15.8)	< 0.001
GLIM aetiologic criteria:				
– Reduced nutrient intake, n (%)	48 (9.2)	35 (24.0)	13 (3.5)	< 0.001
WOMAC total score	54.6 ± 13.8	63.8 ± 12.7	51.0 ± 12.4	< 0.001
WOMAC pain subscale	12.8 ± 3.9	15.1 ± 3.5	11.9 ± 3.7	< 0.001
WOMAC function subscale	37.5 ± 10.4	44.6 ± 9.8	34.8 ± 9.7	< 0.001
Kellgren–Lawrence grade 3–4, n (%)	312 (60.0)	106 (72.6)	206 (55.1)	< 0.001
Length of hospital stay, days	8.9 ± 3.1	10.7 ± 3.6	8.2 ± 2.7	< 0.001
Perioperative complications, n (%)	74 (14.2)	36 (24.7)	38 (10.2)	< 0.001

Regarding the assessment of muscle mass, of the 520 included patients, CT-derived cross-sectional area measurements (quadriceps or psoas) were available in 396 patients (76.2%). Calf circumference was used as the primary muscle mass indicator in 98 patients (18.8%) who lacked usable CT images, and medical-record data were used in the remaining 26 patients (5.0%). A concordance analysis in a subgroup of 135 patients with both CT and calf circumference measurements showed substantial agreement between the two methods (Cohen’s κ = 0.72, 95% CI: 0.63-0.81), supporting the use of calf circumference as a valid supplementary indicator.

Missing data for key variables were minimal, with proportions ranging from 0.8% (BMI) to 5.8% (WOMAC score), all below the 10% threshold for multiple imputation. Specifically, missingness proportions were as follows: serum albumin 4.2%, total lymphocyte count 3.8%, total cholesterol 2.9%, CRP 3.1%, ESR 4.6%, BMI 0.8%, WOMAC score 5.8%, and CT-derived muscle mass 3.5%. Multiple imputation with 5 datasets was performed under the missing-at-random assumption, and complete-case sensitivity analyses confirmed the robustness of the findings.

Among the 520 enrolled patients, the phenotypic criteria were met as follows: involuntary weight loss ( > 5% within 3 months or > 10% within 6 months) was documented in 25 patients (4.8%); low BMI (<18.5 kg/m^2^) was present in 0 patients (0%), as expected given the inclusion criterion of BMI ≥ 28 kg/m^2^; and low muscle mass (by CT-derived CSA or calf circumference) was identified in 138 patients (26.5%). For the aetiologic criteria, reduced nutrient intake ( ≤ 50% of requirement for ≥ 1 week or any reduction for ≥ 2 weeks) was documented in 48 patients (9.2%); and inflammation (CRP ≥ 10 mg/L or documented chronic inflammatory condition) was present in 180 patients (34.6%). A total of 146 patients (28.1%) met the combined requirement of at least one phenotypic criterion and at least one aetiologic criterion and were therefore classified as having GLIM-defined malnutrition.

### Differences in nutritional risk detection rates among different screening tools

Of the seven tools initially screened (NRS-2002, MUST, MNA-SF, MST, GNRI, PNI, and CONUT), MST and PNI were excluded from the final analysis due to redundancy with MUST and CONUT/GNRI, respectively. The final analysis therefore focused on five tools: NRS-2002, MUST, MNA-SF, GNRI, and CONUT.

Different nutritional screening tools showed marked differences in their ability to detect nutritional risk in patients with obesity-related KOA (Figure 2). Overall, BMI-dependent tools had lower detection rates, while laboratory-based tools had relatively higher detection rates (Figure 2A).

**FIGURE 2:**
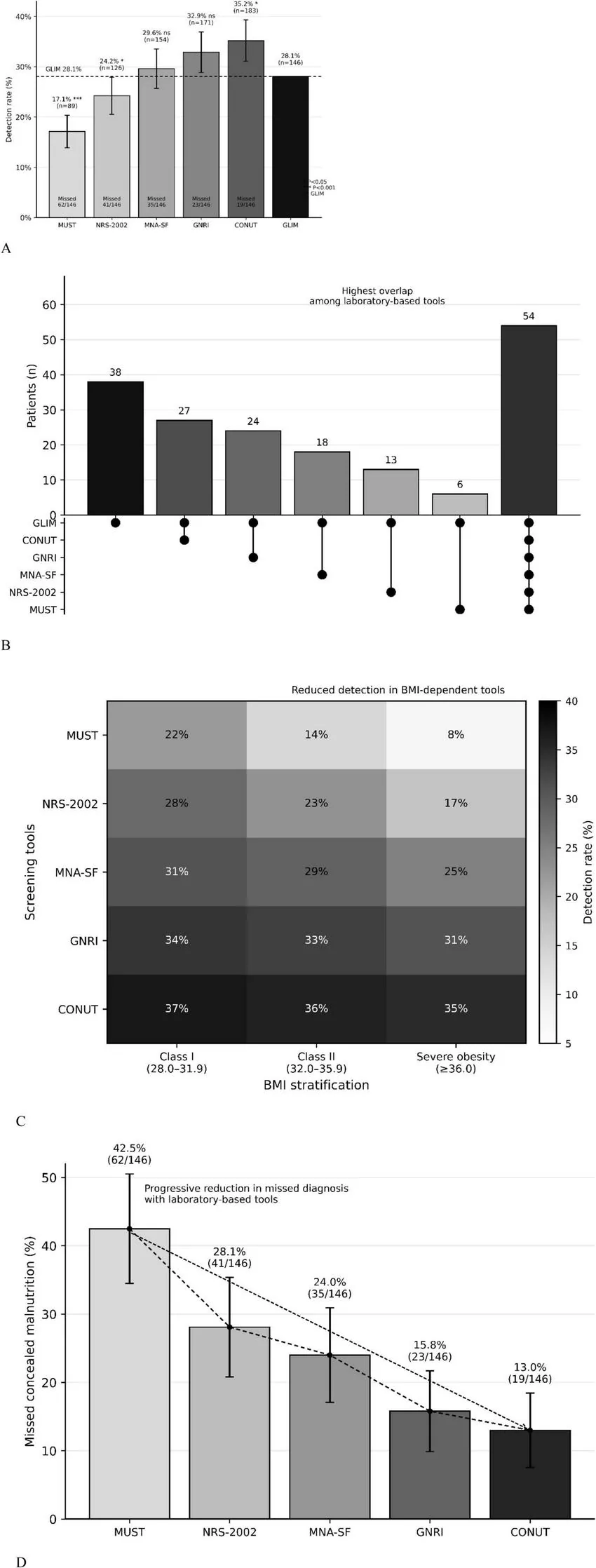
Differences in nutritional risk detection rates and diagnostic agreement among different nutritional screening tools. **(A)** Detection rates of the five screening tools compared with GLIM-defined malnutrition prevalence (28.1%). MUST showed the lowest detection rate (17.1%) and CONUT the highest (35.2%). Pairwise comparisons vs. GLIM were performed using McNemar’s test. **(B)** Venn diagram illustrating the overlap of positive classifications among the five tools and GLIM criteria. **(C)** Detection rates stratified by obesity class (Class I: BMI 28.0–32.4 kg/m^2^; Class II: BMI 32.5–37.4 kg/m^2^; Severe: BMI ≥ 37.5 kg/m^2^). **(D)** Detection rates for concealed malnutrition (defined as GLIM-positive without significant recent weight loss). Error bars represent 95% CIs estimated by bootstrap.

MUST detected nutritional risk in 89 patients, giving a detection rate of 17.1%, which was significantly lower than the GLIM-defined malnutrition prevalence of 28.1% (*P* < 0.001) (Figure 2A). The detection rate of NRS-2002 was 24.2%, while the detection rates of GNRI and CONUT were 32.9 and 35.2%, respectively, with CONUT showing the highest detection rate (Figure 2A). Moreover, MUST had the highest missed diagnosis rate for GLIM-defined malnourished patients (42.5%), whereas CONUT had the lowest (13.0%) (Figure 2A).

Intersection analysis showed that the laboratory-based nutritional indices had higher overlapping recognition ability with each other, whereas the overlap between BMI-oriented tools and GLIM was lower (Figure 2B). As BMI level increased, the detection rates of MUST and NRS-2002 progressively decreased; MUST had a detection rate of only 8% in severely obese patients, markedly lower than the 35% of CONUT (Figure 2C).

Further analysis revealed that BMI-oriented tools tended to substantially underestimate concealed malnutrition, whereas GNRI and CONUT maintained relatively stable detection performance across different BMI strata (Figure 2D). These results suggest that in patients with obesity-related KOA, traditional nutritional screening tools that rely on BMI or weight change may fail to adequately identify true nutritional abnormalities.

### Agreement among nutritional screening tools and diagnostic discordance

Overall agreement among nutritional screening tools was low to moderate (Fleiss’ κ = 0.42). The highest pairwise agreement was between GNRI and CONUT (κ = 0.63), followed by NRS-2002 and MNA-SF (κ = 0.48). Agreement with GLIM was highest for CONUT (κ = 0.58) and weakest for MUST (κ = 0.29) (Table 2). Diagnostic discordance (at least one tool disagreeing) occurred in 37.9% of patients (Table 3). Missed diagnosis rates for GLIM-confirmed malnutrition were highest for MUST (42.5%), followed by NRS-2002 (28.1%), MNA-SF (24.0%), GNRI (15.8%), and lowest for CONUT (13.0%) (Table 3). In obesity-related KOA, traditional BMI-dependent tools may inadequately identify true nutritional abnormalities, whereas laboratory-based indices show higher agreement with GLIM and lower missed diagnosis rates. Scoring ranges, cut-off values, and classification criteria for the five nutritional screening tools is shown in Supplementary Table 1.

**TABLE 2:** Agreement analysis among nutritional screening tools.

Comparison/ Indicator	Statistical measure	Value (95% CI)	Interpretation
NRS-2002 vs. MNA-SF	Cohen’s κ	0.48 (0.41–0.55)	Moderate agreement
GNRI vs. CONUT	Cohen’s κ	0.63 (0.56–0.70)	Substantial agreement
MUST vs. GLIM	Cohen’s κ	0.29 (0.22–0.36)	Fair agreement
GNRI vs. GLIM	Cohen’s κ	0.55 (0.48–0.62)	Moderate agreement
CONUT vs. GLIM	Cohen’s κ	0.58 (0.51–0.65)	Highest agreement with GLIM
Overall agreement among all tools	Fleiss’ κ	0.42 (0.38–0.46)	Moderate overall agreement

**TABLE 3:** Diagnostic discordance analysis among nutritional screening tools.

Screening tool	Missed GLIM-confirmed malnutrition, n/N	Missed diagnosis rate	χ ^2^ value	*P*-value	Comparative interpretation
MUST	62/146	42.5%	31.84	< 0.001	Highest missed diagnosis rate
NRS-2002	41/146	28.1%	12.67	< 0.001	Higher missed diagnosis than laboratory-based tools
MNA-SF	35/146	24.0%	7.95	0.005	Intermediate missed diagnosis rate
GNRI	23/146	15.8%	1.84	0.175	Comparable to CONUT
CONUT	19/146	13.0%	Reference	–	Lowest missed diagnosis rate

Pairwise comparisons of missed diagnosis rates used McNemar’s test for paired data. P values were adjusted for multiple comparisons using the Bonferroni method where applicable.

### Characteristics of concealed malnutrition and sarcopenic obesity

Among the 146 patients with GLIM-defined malnutrition, 121 maintained a BMI still within the obesity range and did not exhibit significant recent involuntary weight loss (i.e., > 5% within 3 months or > 10% within 6 months); these were defined as concealed malnutrition, accounting for 23.3% of the total cohort and 82.9% of GLIM-defined malnourished patients (Figure 3A). The remaining 25 patients (17.1% of GLIM-defined malnourished patients) had significant recent weight loss that met the GLIM phenotypic criterion and were therefore classified as having overt malnutrition, even though their BMI remained ≥ 28 kg/m^2^. This distinction is clinically meaningful: the 25 patients with overt malnutrition had at least one visible warning sign (weight loss), whereas the 121 patients with concealed malnutrition had no low BMI and no weight-loss signal, making their nutritional abnormalities detectable only through muscle mass and inflammatory assessment. These findings indicate that most nutritional abnormalities are markedly concealed in the context of obesity, with traditional BMI- and weight-based indicators failing to capture over 80% of malnutrition cases (Figure 3A).

**FIGURE 3:**
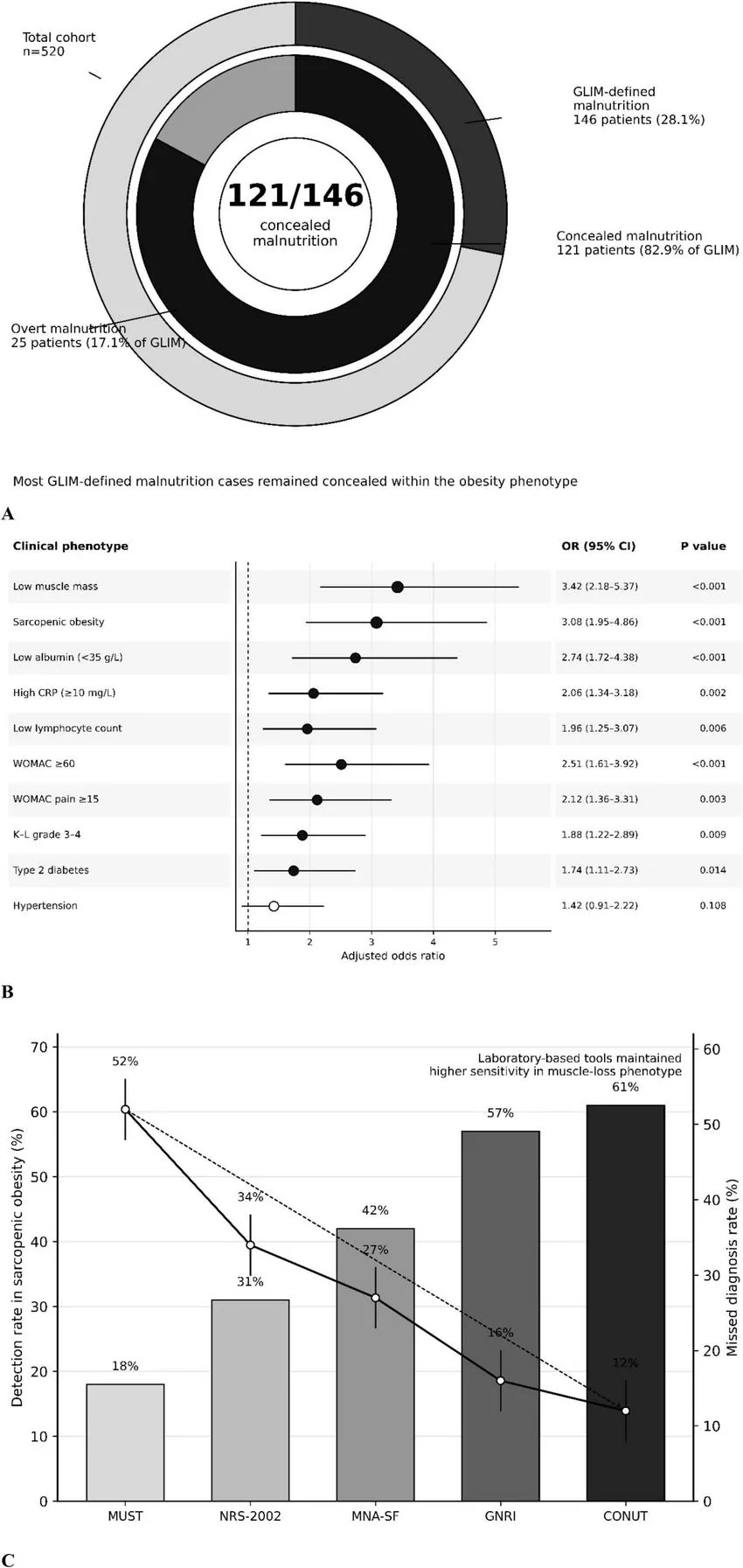
Clinical characteristics of concealed malnutrition and sarcopenic obesity. **(A)** Proportions of concealed malnutrition (121/146, 82.9%) and overt malnutrition (25/146, 17.1%) among GLIM-defined malnourished patients. **(B)** Comparison of inflammatory and muscle-related parameters between concealed malnutrition (*n* = 121) and non-concealed malnutrition (*n* = 399). Statistical comparisons were performed using the Mann–Whitney U test for continuous variables and the χ^2^ test for categorical variables. **(C)** Detection rates of the five screening tools in patients with sarcopenic obesity (*n* = 112). MUST had the lowest detection rate (18%) and CONUT the highest (61%). Error bars represent 95% CIs.

To further clarify the distinction between concealed and overt malnutrition in this obese cohort, we compared the 121 patients with concealed malnutrition and the 25 patients with overt malnutrition (both GLIM-positive with BMI ≥ 28 kg/m^2^). Compared with the concealed malnutrition group, the overt malnutrition group had a significantly higher proportion of recent involuntary weight loss (100% vs. 0%, by definition), lower BMI (29.1 ± 2.4 vs. 31.2 ± 2.7 kg/m^2^, *P* = 0.001), and lower serum albumin (31.8 ± 4.5 vs. 34.6 ± 4.2 g/L, *P* = 0.003). However, no significant differences were observed between the two groups in CRP levels (13.2 vs. 12.6 mg/L, *P* = 0.412), prevalence of low muscle mass (60.0% vs. 73.6%, *P* = 0.178), or WOMAC total score (62.1 ± 13.4 vs. 64.2 ± 12.5, *P* = 0.451). These findings suggest that while weight loss distinguishes overt from concealed presentations, the underlying inflammatory and muscle-wasting components are similarly prevalent in both phenotypes, reinforcing that BMI-based screening alone would miss both groups without systematic GLIM assessment.

Further phenotypic analysis showed that patients with concealed malnutrition had more pronounced features of inflammation activation, muscle loss, and functional impairment (Figure 3B). Low muscle mass showed the strongest association with concealed malnutrition (adjusted OR = 3.42), followed by sarcopenic obesity (OR = 3.08) and hypoalbuminaemia (OR = 2.74) (Figure 3B). High CRP level, higher WOMAC score, and K-L grade 3–4 radiological degeneration were also significantly associated with concealed malnutrition (Figure 3B). In contrast, no significant association was observed between hypertension and concealed malnutrition (*P* = 0.108) (Figure 3B).

A total of 112 patients (21.5% of the overall cohort) had sarcopenic obesity. In this subgroup, the prevalence of GLIM-defined malnutrition reached 58.0%, significantly higher than in patients without sarcopenic obesity. The ability of different screening tools to identify risk in this subgroup differed markedly (Figure 3C). MUST detected nutritional risk in only 18% of patients with sarcopenic obesity, with a missed diagnosis rate as high as 52%, whereas GNRI and CONUT achieved detection rates of 57 and 61%, respectively, with corresponding missed diagnosis rates of only 16 and 12% (Figure 3C). These findings indicate that in the setting of obesity combined with low muscle mass, traditional BMI-oriented screening tools tend to underestimate true nutritional abnormalities, whereas laboratory-based nutritional tools have higher sensitivity for concealed malnutrition.

### Relationship between nutritional risk, inflammatory burden, functional impairment, and radiographic severity

Abnormal nutritional status was significantly associated with chronic inflammation, functional impairment, and structural degeneration (Table 4). Compared with the non-concealed malnutrition group (*n* = 399), patients with concealed malnutrition (*n* = 121) had significantly higher CRP levels (12.6 vs. 6.1 mg/L), higher proportions of high inflammatory burden (51.2% vs. 28.3%), higher neutrophil-to-lymphocyte ratios, and lower serum albumin levels (all *P* < 0.001). They also had substantially higher prevalences of low muscle mass (73.6% vs. 12.3%) and sarcopenic obesity (53.7% vs. 11.8%), as well as worse WOMAC total and subscale scores (all *P* < 0.001) (Table 4). Radiographic severity (K-L grade 3–4: 72.6% vs. 55.1%; bilateral knee involvement: 64.4% vs. 49.2%) and length of hospital stay (9.8 vs. 7.2 days) were also significantly worse in the concealed malnutrition group. Thus, obesity-related concealed malnutrition is a comprehensive clinical phenotype involving inflammation, muscle loss, functional decline, and joint degeneration.

**TABLE 4:** Inflammatory burden, muscle mass, and functional impairment according to concealed malnutrition status.

Indicator	Concealed malnutrition (*n* = 121)	Non-concealed malnutrition (*n* = 399)^†^	*P*-value
CRP, mg/L	12.6 (7.8–19.4)	6.1 (3.5–10.2)	< 0.001
ESR, mm/h	34.8 ± 13.7	23.9 ± 11.4	< 0.001
High inflammatory burden (CRP ≥ 10 mg/L), n (%)	62 (51.2)	113 (28.3)	< 0.001
Neutrophil-to-lymphocyte ratio	3.42 (2.61–4.58)	2.31 (1.76–3.12)	< 0.001
Serum albumin, g/L	34.6 ± 4.2	39.1 ± 3.8	< 0.001
Low muscle mass, n (%)	89 (73.6)	49 (12.3)	< 0.001
Sarcopenic obesity, n (%)	65 (53.7)	47 (11.8)	< 0.001
WOMAC total score	64.2 ± 12.5	50.8 ± 12.3	< 0.001
WOMAC pain subscale	15.3 ± 3.4	11.8 ± 3.6	< 0.001
WOMAC stiffness subscale	5.1 ± 1.7	3.7 ± 1.5	< 0.001
WOMAC function subscale	43.8 ± 9.6	35.3 ± 9.8	< 0.001

† The non-concealed malnutrition group (*n* = 399) includes 374 patients without GLIM-defined malnutrition and 25 patients with overt malnutrition (GLIM-positive with significant recent weight loss).

### Diagnostic performance of nutritional screening tools and reclassification improvement

Using GLIM as the reference, diagnostic performance varied across tools (Figure 4A). CONUT had the highest AUC (0.82), followed by GNRI (0.79), MNA-SF (0.76), NRS-2002 (0.73), and MUST (0.66). CONUT and GNRI significantly outperformed MUST (DeLong’s test), indicating better risk identification with laboratory-based tools. Adding CRP and muscle mass to the traditional model improved the AUC from 0.73 to 0.86 (ΔAUC = 0.13) (Figure 4B). Reclassification analysis showed substantial improvement (NRI = 0.31, IDI = 0.09) (Figure 4C). The base model (traditional model) included age, sex, BMI, hypertension, and type 2 diabetes mellitus, achieving an AUC of 0.73 (95% CI: 0.68–0.77) for predicting GLIM-defined malnutrition. The extended model added CRP (log-transformed) and low muscle mass to the base model, improving the AUC to 0.86 (95% CI: 0.82–0.89), with a ΔAUC of 0.13 (95% CI: 0.09–0.17; P < 0.001). Multicollinearity assessment showed that all VIFs in both models were < 2.0 (range: 1.12–1.78), indicating no significant collinearity among the included predictors. As expected, when sarcopenic obesity (a composite variable defined by low muscle mass plus obesity) was substituted for low muscle mass in the extended model, the VIF exceeded 5.0 (VIF = 6.84), confirming that sarcopenic obesity and low muscle mass are collinear constructs. Therefore, sarcopenic obesity was analyzed separately and was not included together with low muscle mass in the same model. Internal validation using 1,000 bootstrap resamples yielded an optimism-corrected AUC of 0.84 (95% CI: 0.80–0.88) for the extended model, closely matching the apparent AUC of 0.86, indicating low overfitting. The calibration intercept was -0.06 (95% CI: -0.32 to 0.20) and the calibration slope was 0.94 (95% CI: 0.78–1.10), both close to ideal values of 0 and 1, respectively. The Hosmer–Lemeshow test was non-significant (χ^2^ = 8.34, df = 8, *P* = 0.401), and the calibration curve showed good agreement between predicted probabilities and observed outcomes. The Brier score was 0.14, indicating acceptable overall model performance.

**FIGURE 4:**
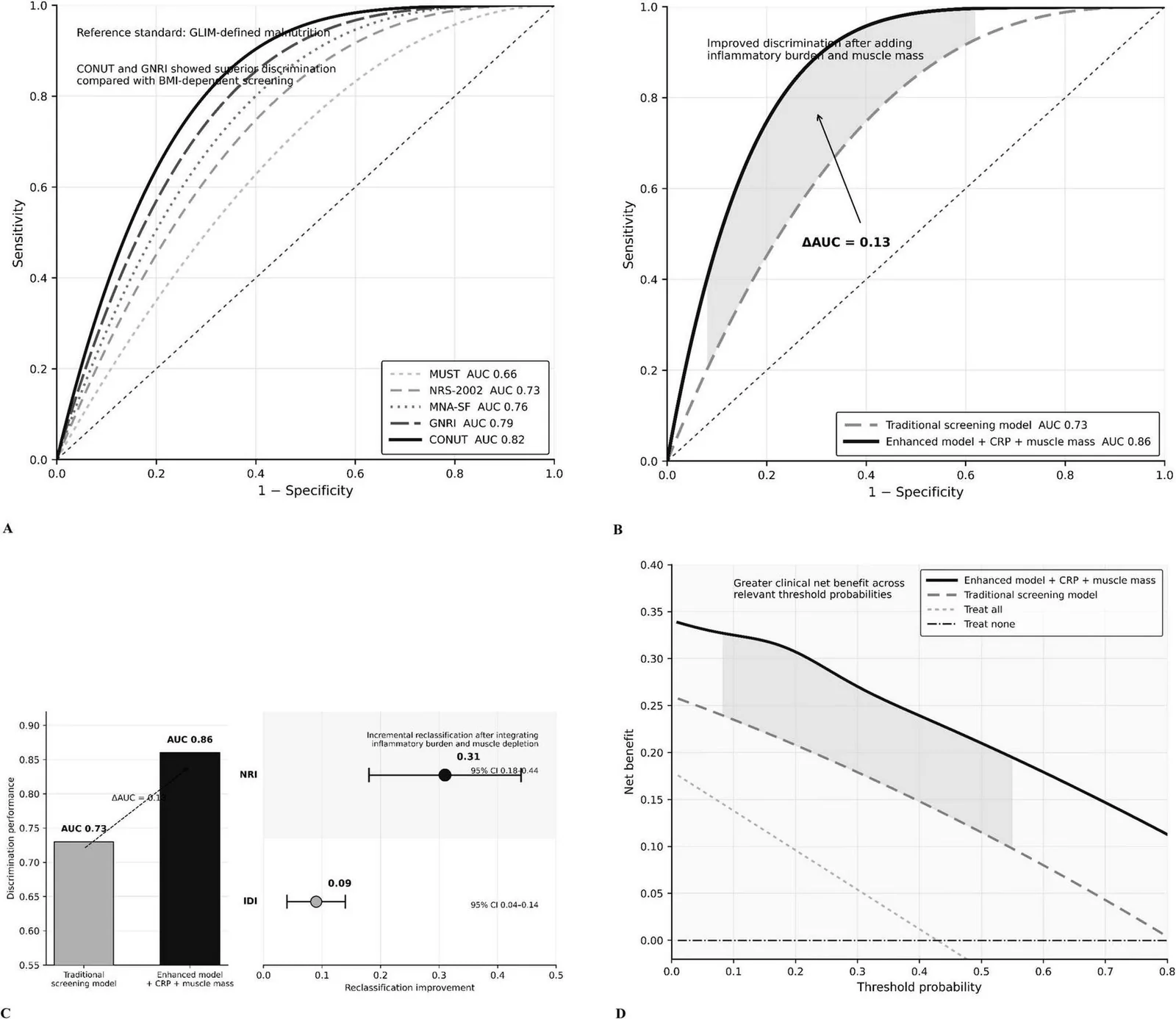
Diagnostic performance and gain analysis of different nutritional screening models. **(A)** Receiver operating characteristic (ROC) curves of the five nutritional screening tools for identifying GLIM-defined malnutrition. CONUT showed the highest AUC (0.82, 95% CI: 0.78–0.86), followed by GNRI (0.79, 95% CI: 0.75–0.83), MNA-SF (0.76, 95% CI: 0.71–0.80), NRS–2002 (0.73, 95% CI: 0.68–0.77), and MUST (0.66, 95% CI: 0.61–0.71). Pairwise comparisons by DeLong’s test showed that CONUT and GNRI significantly outperformed MUST (both *P* < 0.001), but the difference between CONUT and GNRI was not statistically significant (*P* = 0.089). **(B)** ROC curves comparing the base model and the extended model for predicting GLIM-defined malnutrition. The base model (traditional model, dashed line) included age, sex, BMI, hypertension, and type 2 diabetes mellitus (AUC = 0.73, 95% CI: 0.68–0.77). The extended model (solid line) added CRP (log-transformed) and low muscle mass to the base model (AUC = 0.86, 95% CI: 0.82–0.89). The difference was statistically significant by DeLong’s test (*P* < 0.001). **(C)** Net reclassification improvement (NRI) and integrated discrimination improvement (IDI) analysis. The extended model showed significant improvement over the base model: continuous NRI = 0.31 (95% CI: 0.19–0.43) and IDI = 0.09 (95% CI: 0.06–0.12), indicating that incorporating CRP and low muscle mass substantially improved risk classification. (D) Decision curve analysis comparing the base model and the extended model. The extended model (adding CRP and low muscle mass) demonstrated higher net clinical benefit than the base model across a wide range of threshold probabilities (10–80%), indicating that the added variables improve clinical decision-making without increasing unnecessary interventions.

Decision curve analysis confirmed higher net benefit for the combined model across a wide threshold range (Figure 4D). Thus, traditional BMI-dependent screening inadequately identifies true nutritional abnormalities in obesity, whereas a model integrating inflammation and muscle phenotype better captures concealed malnutrition risk.

We calculated the full diagnostic performance metrics for each of the five nutritional screening tools at their prespecified cut-offs [NRS-2002 ≥ 3, MUST ≥ 1 (medium/high risk), MNA-SF ≤ 11 (at risk or malnourished), GNRI ≤ 98 (any grade of risk), CONUT ≥ 2 (any grade of abnormality)]. CONUT achieved the highest sensitivity (87.0%) and the highest negative predictive value (94.4%), indicating its superior ability to rule out malnutrition when negative, whereas MUST showed the lowest sensitivity (57.5%) and the lowest negative predictive value (83.8%). Conversely, MUST had the highest specificity (91.2%), reflecting its tendency to classify fewer patients as positive but at the cost of a high false-negative rate. CONUT also had the highest AUC (0.82, 95% CI: 0.78–0.86), significantly outperforming MUST (AUC = 0.66, 95% CI: 0.61–0.71; DeLong’s test *P* < 0.001). GNRI showed comparable performance to CONUT (AUC = 0.79, 95% CI: 0.75–0.83; P for difference vs. CONUT = 0.089), while MNA-SF and NRS-2002 exhibited intermediate performance (AUC = 0.76, 95% CI: 0.71–0.80 and AUC = 0.73, 95% CI: 0.68–0.77, respectively). The optimal threshold based on the Youden index for each tool and the corresponding performance metrics are also provided in Table 5 and Supplementary Table 2.

**TABLE 5:** Diagnostic performance of each nutritional screening tool against GLIM criteria.

Screening tool	Threshold (risk cut-off)	Sensitivity (%)	Specificity (%)	LR+	LR-	PPV (%)	NPV (%)	AUC (95% CI)	Youden index
NRS-2002	≥ 3	71.9	69.5	2.36	0.41	47.7	86.7	0.73 (0.68–0.77)	0.414
MUST	≥ 1 (medium/high)	57.5	91.2	6.53	0.47	71.2	83.8	0.66 (0.61–0.71)	0.487
MNA-SF	≤ 11 (at risk/malnourished)	76	72.5	2.76	0.33	52.1	88.2	0.76 (0.71–0.80)	0.485
GNRI	≤ 98 (any risk)	84.2	76.2	3.54	0.21	58.6	92.3	0.79 (0.75–0.83)	0.604
CONUT	≥ 2 (any abnormality)	87	73.5	3.28	0.18	57.6	94.4	0.82 (0.78–0.86)	0.605

To evaluate whether the improved AUC and reclassification metrics were merely tautological, we performed the two prespecified sensitivity analyses. First, within the concealed malnutrition subgroup (*n* = 121), where GLIM positivity was exclusively determined by low muscle mass and/or inflammation (since weight loss and low BMI were definitionally absent), the extended model still showed excellent discrimination (AUC = 0.85, 95% CI: 0.79–0.91), confirming that these variables effectively capture the core pathophysiological features driving this phenotype. Second, when the GLIM reference was redefined using only weight loss and reduced intake (i.e., excluding low muscle mass and inflammation from the reference standard), the addition of CRP and low muscle mass to the base model still improved the AUC from 0.72 (95% CI: 0.67–0.77) to 0.81 (95% CI: 0.76–0.86), with NRI = 0.24 and IDI = 0.07. These findings indicate that the incremental diagnostic value of incorporating CRP and muscle mass is not wholly attributable to criterion overlap with the GLIM reference, but rather reflects genuine improvement in identifying at-risk patients who would otherwise be missed by traditional BMI-dependent screening.

### Independent correlates of concealed malnutrition and model stability

Multivariable regression identified low muscle mass, high inflammatory burden, and functional impairment as independent correlates of concealed malnutrition (Figure 5A and Table 6). After adjustment, low muscle mass (OR = 3.42, 95% CI: 2.18-5.37), sarcopenic obesity (OR = 2.88, 95% CI: 1.84-4.52), high CRP, hypoalbuminaemia, and higher WOMAC score remained significant (all *P* < 0.01) (Figure 5A and Table 6). A non-linear relationship was observed between CRP and concealed malnutrition risk, with accelerated risk increase after ∼8–10 mg/L (P for nonlinearity = 0.018) (Figure 5B and Table 6). Internal validation (1,000 bootstrap) showed good calibration (Brier score = 0.14) (Figure 5C and Table 6). Sensitivity analyses confirmed robustness (Figure 5D and Table 6). Thus, concealed malnutrition in obesity-related KOA is a complex metabolic-nutritional phenotype driven by inflammation, muscle loss, and functional decline, not merely BMI abnormalities.

**FIGURE 5:**
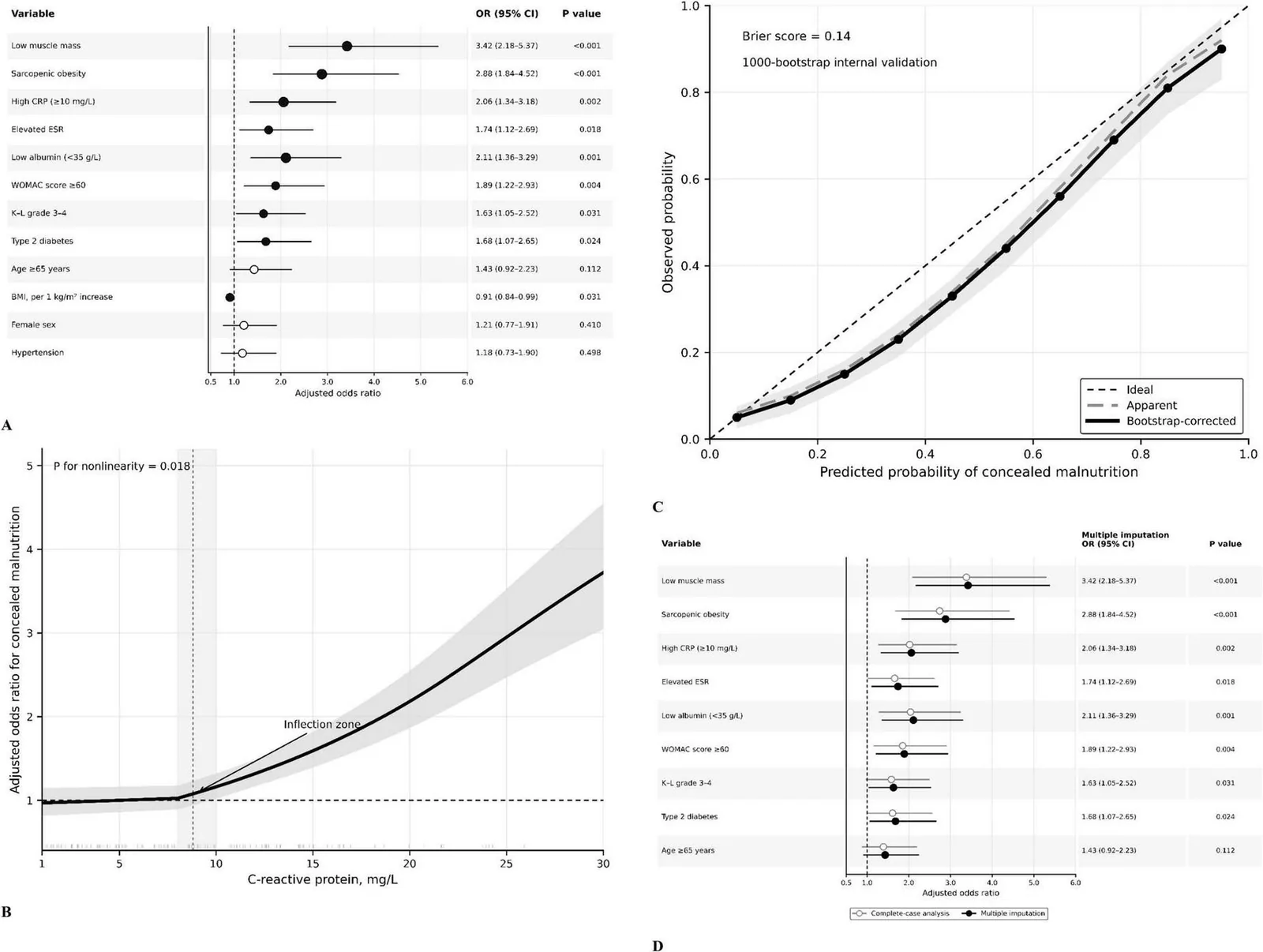
Independent correlates of concealed malnutrition and model stability analysis. **(A)** Multivariable logistic regression forest plot. All variables were entered simultaneously (forced entry). Low muscle mass and sarcopenic obesity were evaluated in separate models to avoid collinearity (VIFs for all predictors were < 2.0). Adjusted ORs with 95% CIs are shown. **(B)** Restricted cubic spline analysis of the association between CRP level and concealed malnutrition risk. The nonlinear association was significant (P for nonlinearity = 0.018). **(C)** Calibration curve from 1,000 bootstrap internal validation of the extended model. Dashed line represents ideal calibration; solid line shows observed performance (calibration intercept = −0.06, 95% CI: −0.32 to 0.20; calibration slope = 0.94, 95% CI: 0.78–1.10; Brier score = 0.14; Hosmer-Lemeshow *P* = 0.401). **(D)** Sensitivity analysis comparing complete-case analysis and multiple imputation; all effect estimates remained directionally consistent.

**TABLE 6:** Multivariable regression, nonlinear association, and internal validation analyses for concealed malnutrition.

Analysis domain	Variable/ assessment	Adjusted OR or result	95% CI	*P*-value
Multivariable regression	Low muscle mass	3.42	2.18–5.37	< 0.001
	Sarcopenic obesity	2.88	1.84–4.52	< 0.001
	High CRP ( ≥ 10 mg/L)	2.06	1.34–3.18	0.002
	Elevated ESR	1.74	1.12–2.69	0.018
	Low albumin ( < 35 g/L)	2.11	1.36–3.29	0.001
	WOMAC score ≥ 60	1.89	1.22–2.93	0.004
	K-L grade 3–4	1.63	1.05–2.52	0.031
	Type 2 diabetes	1.68	1.07–2.65	0.024
	Age ≥ 65 years	1.43	0.92–2.23	0.112
	BMI, per 1 kg/m^2^ increase	0.91	0.84–0.99	0.031
Restricted cubic spline analysis	CRP nonlinear association	Risk acceleration beyond 8–10 mg/L	–	P for nonlinearity = 0.018
Bootstrap internal validation	Calibration performance	Brier score = 0.14	1000-bootstrap resampling	–
Sensitivity analysis	Complete-case vs. multiple imputation	Consistent effect estimates	Stable directionality	–

OR, odds ratio; CI, confidence interval; VIF, variance inflation factor; NRI, net reclassification improvement; IDI, integrated discrimination improvement.

To assess whether the use of supplementary calf-circumference data introduced measurement bias, we repeated the main diagnostic agreement and model performance analyses in the subgroup of 396 patients (76.2% of the cohort) whose muscle mass was assessed exclusively by CT (quadriceps or psoas CSA). In this CT-only subgroup, the prevalence of GLIM-defined malnutrition was 29.3% (116/396), comparable to the full-cohort prevalence of 28.1% (146/520). The agreement between CONUT and GLIM remained similar (κ = 0.59, 95% CI: 0.52–0.66) to that in the full cohort (κ = 0.58, 95% CI: 0.51–0.65), and MUST continued to show the lowest agreement (κ = 0.30, 95% CI: 0.23–0.37). The extended model incorporating CRP and low muscle mass achieved an AUC of 0.85 (95% CI: 0.80–0.90) in the CT-only subgroup, closely matching the full-cohort AUC of 0.86. The reclassification metrics in the CT-only subgroup were also consistent (NRI = 0.30, IDI = 0.08). These findings indicate that the inclusion of calf-circumference data did not materially alter the primary conclusions, and the diagnostic performance and model improvement were robust across different methods of muscle mass assessment.

## Discussion

The term “concealed malnutrition” used in this study is conceptually similar to the “hidden malnutrition” described by Vieira et al. (11) and Mwala et al. (25). However, we deliberately use “concealed” to emphasize the mechanism of BMI masking, that is, a high BMI value (still in the obese range) conceals underlying protein depletion, muscle loss, and inflammation-driven catabolism, leading to systematic under-recognition by traditional BMI-dependent tools. This mechanistic emphasis on BMI as a confounder distinguishes our definition from the broader concept in the literature. The distinction between concealed and overt malnutrition within this obese cohort was operationalized by the presence or absence of significant recent involuntary weight loss. This distinction is clinically relevant because patients without weight loss have no visible anthropometric warning sign, making their malnutrition truly “concealed” by high BMI. In contrast, even when BMI remains in the obese range, weight loss provides an observable clinical cue that should prompt nutritional evaluation. By separating these two groups, we aimed to specifically evaluate the performance of screening tools in the most challenging scenario, where neither low BMI nor weight loss alerts the clinician to underlying malnutrition.

Focusing on this clinical problem in patients with obesity-related KOA, we systematically compared the diagnostic agreement of different nutritional screening tools with GLIM and evaluated the added value of inflammatory burden and muscle mass for nutritional risk identification. The results showed that traditional BMI-dependent tools have a marked tendency to miss malnutrition in this population, whereas a comprehensive assessment strategy integrating laboratory nutritional indices, inflammatory status, and muscle mass more effectively identifies true nutritional abnormalities. Moreover, concealed malnutrition was closely associated with chronic inflammation, functional impairment, and sarcopenic obesity, suggesting that nutritional abnormalities in obesity are not simply an energy imbalance but rather a complex metabolic-inflammatory phenotype. These findings further emphasize that relying solely on BMI or weight change for nutritional screening in these patients may not accurately reflect true nutritional reserve status.

One of the most important findings is the marked heterogeneity in diagnostic results among different tools. The nutritional risk detection rates of CONUT and GNRI were significantly higher than that of MUST, and agreement between MUST and GLIM was the lowest (κ = 0.29), suggesting that traditional BMI-oriented tools may systematically underestimate true nutritional risk in obese patients. This result is consistent with recent research on obesity-related malnutrition. A perioperative study in 2024 indicated that traditional screening tools relying on BMI and weight loss have substantially reduced sensitivity in obese patients, whereas composite tools incorporating laboratory indices can identify high-risk patients earlier (26). Another 2023 study of hospitalized obese patients found that a high BMI does not exclude malnutrition; rather, inflammatory burden and muscle loss are often important determinants of adverse outcomes (27). The higher agreement of CONUT and GNRI may relate to their ability to simultaneously reflect protein reserves, immune status, and metabolic-inflammatory burden. Previous studies have shown that albumin, lymphocyte count, and cholesterol levels not only reflect nutritional status but are also closely related to chronic inflammation and immune-metabolic dysregulation (28). In patients with obesity-related KOA, continuous release of inflammatory cytokines from adipose tissue can increase protein catabolism and inhibit muscle synthesis without overt weight loss, making it more easily overlooked by traditional tools (29). MUST’s missed diagnosis rate of 42.5% further indicates the substantial limitations of BMI-dependent tools in obese patients.

Another important finding is the high proportion of concealed malnutrition in obesity-related KOA and its significant association with sarcopenic obesity and inflammatory burden. More than 80% of GLIM-defined malnourished patients still had a BMI within the obesity range, indicating that a high BMI does not represent adequate nutritional reserves. This finding supports the recently proposed concept of “metabolic malnutrition,” in which obese patients can have both fat accumulation and protein depletion (30). The 2024 international expert consensus on sarcopenic obesity stated that sarcopenic obesity is not only a body composition abnormality but also a high-risk state accompanied by inflammation activation, insulin resistance, and immune dysfunction (31). In this study, the prevalence of GLIM-defined malnutrition was significantly higher in patients with sarcopenic obesity, and MUST had the most pronounced missed diagnosis in this subgroup, further illustrating the difficulty of traditional screening models in identifying nutritional risk caused by muscle loss. Previous studies have shown that activity limitation in osteoarthritis can lead to disuse atrophy of skeletal muscle, while chronic low-grade inflammation promotes muscle protein degradation and mitochondrial dysfunction (32). Moreover, infiltration of adipose tissue into skeletal muscle in the obese state can cause a parallel decline in muscle mass and function, forming the specific phenotype of sarcopenic obesity (33). These results suggest that in obesity-related KOA, low muscle mass may be a better indicator of true nutritional abnormality and functional risk than BMI.

Notably, we found consistently directional associations between concealed malnutrition and inflammatory burden, functional impairment, and radiographic severity. CRP levels were significantly higher in the GLIM-malnourished group, and the proportion of Kellgren-Lawrence grade 3–4 was higher, suggesting that nutritional abnormalities may progress in parallel with structural joint degeneration. Increasingly, studies have suggested that obesity-related osteoarthritis is essentially a low-grade chronic inflammatory disease. Mechanistic studies from 2023 showed that inflammatory mediators such as IL-6, TNF-α, and CRP are not only involved in cartilage degradation but also promote skeletal muscle proteolysis and dysregulation of appetite regulation (34). In chronic inflammation, the body can remain catabolic, and even if body weight remains stable, loss of muscle mass and protein reserves may occur (35). Restricted cubic spline analysis further showed that the risk of concealed malnutrition increased markedly when CRP levels exceeded approximately 8–10 mg/L, suggesting a threshold effect of chronic inflammation in obesity*related nutritional abnormalities. Similar non-linear relationships between high CRP levels and the risk of malnutrition and sarcopenia have been reported in cancer and chronic kidney disease (36). This study extends this phenomenon to obesity-related KOA, indicating that inflammatory burden may be an important biological link connecting joint degeneration, muscle loss, and nutritional abnormalities.

This study also found that adding CRP and muscle mass to the traditional screening model significantly improved diagnostic performance and risk reclassification. The AUC of the combined model increased from 0.73 to 0.86, and both NRI (0.31) and IDI (0.09) showed substantial improvement, suggesting that inflammation and muscle mass information can effectively identify high-risk patients missed by traditional tools. This finding aligns with recent developments in precision nutritional assessment. A 2024 study on GLIM application pointed out that future nutritional assessments should integrate inflammation, biomarkers, and body composition to establish disease-specific risk identification frameworks (37). In the perioperative orthopedic field, low muscle mass and high inflammatory burden are closely related to infection, prosthetic complications, and delayed functional recovery (38). This study further suggests that nutritional risk in obesity-related KOA may reflect an inflammation-muscle-metabolism coupling abnormality rather than simple inadequate energy intake. Therefore, compared with traditional BMI-oriented screening, a comprehensive assessment strategy integrating inflammation and muscle mass indices may better reflect the true pathophysiological characteristics of obesity-related osteoarthritis.

A methodological concern that warrants explicit discussion is the potential circularity arising from using CRP and low muscle mass, which are GLIM components, as predictors of GLIM-defined malnutrition. We fully acknowledge this conceptual overlap. However, the principal aim of this analysis was not to discover new independent biomarkers, but to quantify the real-world diagnostic gain when two frequently under-measured GLIM elements (inflammation reflected by CRP and muscle mass) are systematically incorporated into traditional BMI-dependent screening. In many clinical settings, CT-derived muscle mass and CRP are not routinely measured as part of nutritional screening. Their omission leads to substantial underdiagnosis of malnutrition, particularly in obese patients where BMI is misleadingly normal or elevated. The observed AUC improvement (from 0.73 to 0.86) therefore represents the incremental benefit of operationalizing the full GLIM framework with objective measurements, rather than a tautological confirmation of GLIM against itself.

Importantly, the sensitivity analyses described above support this interpretation. When we restricted the analysis to the concealed malnutrition subgroup (where GLIM positivity depended entirely on low muscle mass and/or inflammation), the extended model maintained excellent discrimination (AUC = 0.85). Moreover, when we redefined the GLIM reference to minimize overlap with the added predictors (using only weight loss plus reduced intake), the addition of CRP and muscle mass still improved reclassification (AUC from 0.72 to 0.81; NRI = 0.24). These results suggest that while some degree of mathematical overlap is inherent, the incremental value is not spurious. Nevertheless, readers should interpret the reclassification metrics with the understanding that partial overlap exists, and future external validation studies using fully independent objective measures, such as dual-energy X-ray absorptiometry or bioelectrical impedance analysis for muscle mass and alternative inflammatory markers such as IL-6, are warranted to confirm these findings.

Furthermore, these results have clinical translational implications. Current perioperative orthopedic management increasingly emphasizes enhanced recovery and precise risk stratification, but obese patients are often assumed to have adequate nutritional reserves by default, thus neglecting their potential nutritional risk (39). We found that patients with concealed malnutrition not only had higher inflammatory burden but also worse WOMAC scores, suggesting that nutritional abnormalities may already be involved in functional deterioration. Existing studies have shown that perioperative nutritional support, protein supplementation, and exercise interventions can improve postoperative recovery and functional outcomes in patients with sarcopenic obesity (40). Therefore, preoperative nutritional assessment should address muscle quality, inflammatory burden, and immune-nutritional status, not just weight control. The inflammation-muscle mass combined assessment framework proposed here may offer a new path for precise perioperative nutritional intervention.

Based on these observations, we offer several preliminary and hypothesis-generating considerations for clinical practice, with the caveat that they derive from a single-center retrospective study and require prospective validation. First, the high missed diagnosis rate (42.5%) and poor agreement with GLIM (κ = 0.29) suggest that MUST may have limited sensitivity in this obese population. In contrast, CONUT and GNRI showed substantially lower missed diagnosis rates (13.0 and 15.8%, respectively) and higher agreement with GLIM, suggesting that laboratory-based indices might be more suitable for screening in obesity-related KOA. However, these observations should not be interpreted as an unequivocal recommendation to avoid MUST or universally adopt CONUT and GNRI without further validation. Second, even when screening tools return negative results, a formal nutritional assessment may be warranted in patients with BMI ≥ 28 kg/m^2^ if any of the following features are present: CRP level approaching 8–10 mg/L, low muscle mass (or clinical signs such as reduced calf circumference), or a WOMAC total score of 60 or higher (indicating severe functional limitation). These thresholds are provisional and require external validation. Third, a pragmatic two-step strategy could serve as a working framework for future evaluation. Step 1 uses CONUT with a cut-off of 2 or higher to detect any abnormality, and step 2 confirms the diagnosis using GLIM criteria, with particular emphasis on objective assessment of muscle mass (preferably by CT, or by calf circumference when CT is unavailable) and inflammatory burden (CRP ≥ 10 mg/L). We emphasize that this framework is provisional and should be tested in prospective multicenter cohorts before implementation in routine clinical practice.

This study has several limitations. First, it is a single-center retrospective study, which may have selection bias, and the generalizability of the results to community or outpatient populations needs further validation. Second, muscle mass assessment in some patients relied on CT cross-sectional area analysis, and there may be heterogeneity between different imaging levels and measurement methods. Third, this study focused mainly on cross-sectional associations and cannot establish causality between concealed malnutrition and osteoarthritis progression. Future multicenter prospective cohort studies, integrating metabolomics, inflammatory cytokine profiles, and dynamic changes in body composition, are needed to further elucidate the biological mechanisms and long-term clinical significance of concealed malnutrition in obesity-related KOA.

## Conclusion

In this single-center retrospective cohort of patients with obesity-related KOA, the prevalence of GLIM-defined malnutrition was 28.1%, with more than 80% of malnourished patients exhibiting concealed malnutrition that was masked by high BMI and not accompanied by weight loss. Traditional BMI-dependent screening tools, particularly MUST, demonstrated the highest missed diagnosis rate and the poorest agreement with GLIM, whereas CONUT showed the best diagnostic performance in our cohort. Incorporating CRP and low muscle mass into the traditional model substantially improved risk reclassification, highlighting the pragmatic value of operationalizing the full GLIM framework in this population. Low muscle mass, high CRP, and worse functional status were independently associated with concealed malnutrition. These findings suggest that laboratory-based nutritional indices may be more suitable for screening in obesity-related KOA and that systematic assessment of inflammation and muscle mass could improve risk stratification. However, these conclusions are hypothesis-generating and based on a single-center retrospective design. Prospective multicenter external validation studies are essential before any definitive changes to clinical practice can be recommended.

## Materials and methods

### Patient characteristics

#### Study design

This was a single-center, retrospective, cross-sectional diagnostic agreement study using data from Shanghai Sixth People’s Hospital. Consecutive patients hospitalized for KOA between January 2022 and December 2024 were enrolled.

During the study period, 1,012 hospitalized patients were initially screened. After applying the prespecified inclusion and exclusion criteria, 364 patients were excluded for the following reasons: BMI < 28.0 kg/m^2^ (*n* = 223), not primary knee osteoarthritis (*n* = 72), outpatient or day-surgery (*n* = 45), and bilateral stage ≥ IV requiring immediate revision (*n* = 24). A further 128 were excluded due to inflammatory or autoimmune disease (*n* = 34), active malignancy (*n* = 29), chronic liver or kidney failure (*n* = 26), acute infection within 2 weeks (*n* = 21), prior knee replacement (*n* = 10), or incomplete data (*n* = 8). Of the remaining 648 patients, seven nutritional screening tools were initially assessed during data collection (NRS-2002, MUST, MNA-SF, MST, GNRI, PNI, and CONUT); however, MST and PNI were subsequently excluded from the final analysis due to redundancy and lack of specific validation in this population. Finally, 520 patients were included in the statistical analysis.

All data were obtained from the hospital’s electronic medical record system, laboratory information system, and picture archiving and communication system. Case screening and data verification were performed independently by two uniformly trained researchers; any disagreements were reviewed and adjudicated by a senior researcher to ensure consistency and reliability.

All clinical data were extracted from the patient’s first hospitalization, and laboratory tests and clinical assessments completed within 24 h of admission were used as baseline data to minimized the potential impact of in-hospital treatment and disease progression on nutritional status evaluation.

This study strictly followed the Declaration of Helsinki and international ethical guidelines for medical research and was approved by the Medical Ethics Committee (WJEC-YJS-2024-014-P001).

### Inclusion and exclusion criteria

#### Inclusion criteria

Patients were included if they met all of the following criteria: (1) age ≥ 40 years; (2) meeting the American College of Rheumatology (ACR) diagnostic criteria for knee osteoarthritis; (3) radiographic evidence of degenerative changes in the knee with Kellgren-Lawrence (K-L) grading available; (4) body mass index (BMI) meeting the definition of obesity (BMI ≥ 28 kg/m^2^, according to Chinese adult obesity criteria); (5) nutritional indices, including serum albumin, total lymphocyte count, total cholesterol, and body weight data, completed within 24 h of admission during the first hospitalization; (6) complete medical records allowing calculation of all nutritional screening tools; (7) complete functional and laboratory data, including pain scores, inflammatory markers, and radiographic assessments.

#### Exclusion criteria

Patients were excluded if any of the following were present: (1) active malignant tumor; (2) active infection, autoimmune disease, or acute trauma; (3) end-stage liver failure, end-stage kidney disease, or severe cardiac dysfunction; (4) major surgery or systematic nutritional support within the previous 3 months; (5) severe edema, ascites, or obvious fluid retention affecting the accuracy of body weight measurement; (6) long-term use of glucocorticoids or immunosuppressants; (7) cognitive dysfunction or psychiatric disease preventing completion of assessments; (8) missing > 20% of key variables; (9) repeat hospitalizations (only the first complete episode was retained).

### Study methods and grouping

#### Data source and baseline data extraction

All data for included patients were extracted from the electronic medical record system, laboratory information system, and picture archiving and communication system during the first hospitalization. Using a prespecified case report form, researchers extracted demographic information, anthropometric measurements, medical history, admission diagnoses, treatment modalities, and routine examinations completed during the first hospitalization. All baseline data were based on the first recorded values within 24 h of admission to minimize the influence of in-hospital treatment on baseline status.

Demographic and clinical data included age, sex, height, weight, BMI, smoking history, alcohol use, and comorbid conditions. Comorbidities included hypertension, type 2 diabetes mellitus, coronary artery disease, and chronic kidney disease, confirmed by documented diagnoses, long-term medication records, or discharge diagnoses. BMI was calculated as weight divided by height squared and was used only for baseline description and stratification.

#### Nutritional screening and nutritional status assessment process

A multi-dimensional nutritional assessment framework was used, and each patient was assessed with NRS-2002, MUST, MNA-SF, GNRI, and CONUT. The information required for each tool was obtained from medical records, anthropometric measurements, and first laboratory tests obtained within 24 h of admission. Nutritional scores were independently calculated by two uniformly trained researchers; before scoring, the definitions, variable sources, and calculation methods were standardized. Any disagreement was resolved by re-examining the original medical records and reaching a consensus with a third researcher.

Of the seven nutritional assessment tools initially considered (NRS-2002, MUST, MNA-SF, MST, GNRI, PNI, and CONUT), five were selected for the final analysis (NRS-2002, MUST, MNA-SF, GNRI, and CONUT). MST was excluded because it is primarily a rapid screening tool for identifying patients who need further assessment and lacks a validated cut-off for risk stratification in chronic inflammatory conditions; moreover, it showed high redundancy with MUST (Spearman’s rho = 0.79) in a preliminary correlation analysis. PNI was excluded because it is primarily validated in gastrointestinal and hepatic disease populations, and its component variables (albumin and lymphocyte count) are already captured by CONUT and GNRI; preliminary analysis showed near-identical classification to CONUT (Cohen’s κ = 0.91). To avoid redundancy and multiple-testing inflation, we retained the five most clinically and conceptually distinct tools. The initial screening framework including all seven tools is shown in the study flowchart (Figure 1), while the final analytical results focus on the five selected tools.

#### Identification of concealed malnutrition

Because BMI may mask true nutritional abnormalities in obese patients, a specific process for identifying concealed malnutrition was established in addition to conventional nutritional screening. All patients first underwent nutritional screening with the five tools, and then malnutrition status was further confirmed using the GLIM framework. Data required for the GLIM assessment, including records of recent involuntary weight loss, muscle mass information, changes in nutrient intake, and clinical information related to inflammation, were extracted from the original medical records and examination reports of the first hospitalization. Muscle mass information was preferentially obtained from lower-limb or lumbar CT images available from the hospital stay. CT measurements were performed by two trained researchers using the same image analysis software for muscle cross-sectional area delineation, with uniform window width, window level, and anatomical levels. For patients without usable CT data, calf circumference or nutritional assessment records from the medical records were used as Supplementary Information.

#### Grouping and subgroup construction

Analytical groups were constructed based on nutritional assessment results and clinical characteristics. First, patients were divided into a malnourished group and a non-malnourished group according to the GLIM assessment. Second, based on the presence or absence of malnutrition in the setting of obesity, patients were further classified into concealed malnutrition and non-concealed malnutrition groups. To evaluate the influence of different clinical backgrounds on nutritional risk identification, subgroups were also constructed according to BMI level, age, inflammatory status, muscle mass status, and presence of type 2 diabetes. The specific definitions for BMI stratification, inflammatory status stratification, sarcopenic obesity, and nutritional risk grades are uniformly described.

#### Data quality control

Before the study began, a uniform data extraction specification and variable dictionary were established, clearly defining the source, extraction time point, and entry format for each variable. All researchers completed consistency training before formal data extraction. After data entry, another researcher performed cross-checking, and 10% of the cases were randomly selected for item-by-item comparison with the original medical records to verify accuracy. For obvious outliers, the researchers re-checked the original medical records, laboratory reports, or imaging data. For patients with repeat hospitalizations, only the first hospitalization that met the criteria and had the most complete data was retained.

### Outcome measures

#### Nutritional risk identification and diagnostic discordance metrics

(1) NRS-2002 (range 0–7) evaluates nutritional risk based on nutritional status impairment, disease severity, and age adjustment. A score < 3 indicates no clear risk, ≥ 3 indicates risk. Because NRS-2002 incorporates disease burden, this study focused on its ability to identify nutritional abnormalities in chronic inflammation.

(2) MUST (range 0–6) is based on BMI, recent involuntary weight loss percentage, and acute illness effect. Score 0 = low risk, 1 = medium risk, ≥ 2 = high risk. Because MUST strongly depends on BMI, we evaluated whether it underestimates nutritional risk in obese patients.

(3) MNA-SF (range 0–14) evaluates appetite change, mobility, recent weight change, and neuropsychological status. Scores 12–14 = normal, 8–11 = at risk, 0–7 = malnourished. The study focused on its ability to identify function-limited nutritional abnormalities.

(4) GNRI evaluates protein reserves based on serum albumin, current weight, and ideal weight. Calculation: GNRI = 1.489 × albumin (g/L) + 41.7 × (current/ideal weight), ratio capped at 1 if current > ideal. GNRI > 98 = no risk, 92–98 = mild, 82–91 = moderate, < 82 = severe.

(5) CONUT (range 0–12) evaluates protein reserves, lipid metabolism, and immune-nutritional status using albumin, total cholesterol, and lymphocyte count. 0–1 = normal, 2–4 = mild, 5–8 = moderate, 9–12 = severe. Because CONUT is laboratory-based, we focused on its ability to identify concealed malnutrition in high-BMI patients.

(6) GLIM criteria were used as the reference standard for malnutrition diagnosis. A diagnosis of malnutrition required at least one phenotypic criterion and at least one aetiologic criterion. The operational definitions for each criterion, based on the GLIM consensus and adapted to our retrospective data sources, were as follows:

*Phenotypic criteria:*

- Low BMI: BMI < 18.5 kg/m^2^, using Asian-specific cut-offs recommended by the WHO for Asian populations. Since all enrolled patients met the inclusion criterion of BMI ≥ 28 kg/m^2^, the low BMI criterion was met by none (*n* = 0) in this cohort.
- Involuntary weight loss: Documented unintentional weight loss of > 5% within the preceding 3 months or > 10% within the preceding 6 months, extracted from the admission medical history records (patient-reported or caregiver-reported) and cross-verified with available prior hospital or clinic weight records when accessible.
- Low muscle mass: Assessed primarily by CT-derived cross-sectional areas (CSAs). The CT images used for measurement were obtained from routine clinical examinations (lower-extremity or lumbar CT) performed during the index hospitalization. For CT-derived muscle mass assessment, the quadriceps cross-sectional area (CSA) was measured at the mid-thigh level (defined as the midpoint between the superior border of the patella and the anterior superior iliac spine) on a single axial slice at that level. The measurement was performed on the symptomatic or operative leg for patients with unilateral KOA; for patients with bilateral KOA, the leg with more severe symptoms (higher WOMAC pain score) was selected for consistency. The psoas CSA was measured at the L3 vertebral level (defined as the axial slice at the transverse process of L3) on a single axial slice, and the measurement was performed on the right side in all patients to ensure standardization, as previous studies have shown no significant side-to-side difference in psoas CSA in the absence of unilateral pathology. A patient was classified as having low muscle mass if either the quadriceps CSA or psoas CSA fell below the respective sex-specific cut-off. For patients without usable CT data, calf circumference was measured using a non-elastic tape measure at the maximal circumference of the calf (the widest point between the knee and ankle). The measurement was performed on both legs, and the mean value of the two legs was used for analysis to account for potential side-to-side differences. Patients were in the supine position with the knee relaxed and extended during measurement, following the standardized protocol recommended by the Asian Working Group for Sarcopenia (AWGS). Measurements were taken twice on each leg, and the average was recorded.

Measurements were performed independently by two trained researchers using a dedicated image-analysis workstation with uniform window width, window level, and anatomical levels. The specific muscle groups, anatomical levels, and sex-specific cut-offs were as follows:

Quadriceps CSA: measured at the mid-thigh level (defined as the midpoint between the superior border of the patella and the anterior superior iliac spine). Cut-offs for low muscle mass: < 30.0 cm^2^ for males and < 20.0 cm^2^ for females, based on previously validated Asian reference data. Psoas CSA: measured at the L3 vertebral level (defined as the axial slice at the transverse process of L3). Cut-offs for low muscle mass: < 6.0 cm^2^/m^2^ for males and < 4.5 cm^2^/m^2^ for females (normalized by height^2^), based on published Asian population reference ranges.

A patient was classified as having low muscle mass if either the quadriceps CSA or psoas CSA fell below the respective sex-specific cut-off. When CT data were unavailable (e.g., patients who did not undergo CT imaging during admission), calf circumference was used as a secondary indicator, with cut-offs of < 34 cm for males and < 33 cm for females, consistent with the Asian Working Group for Sarcopenia (AWGS) 2019 criteria. Of the 520 included patients, CT images suitable for muscle area quantification were available in 396 patients (76.2%), comprising 284 patients with mid-thigh CT covering the quadriceps and 112 patients with lumbar CT covering the L3 psoas (some patients had both). Calf circumference was used as the primary muscle mass indicator in 98 patients (18.8%) who lacked usable CT images. In the remaining 26 patients (5.0%), muscle mass information was extracted from documented nutritional assessment notes in the medical records (e.g., prior sarcopenia screening results or anthropometric measurements recorded by dietitians), without quantitative imaging or circumference data. To ensure consistency between CT-based and calf-circumference-based classifications, we performed a concordance analysis in a subgroup of 135 patients who had both CT and calf circumference measurements available; the agreement between the two methods was substantial (Cohen‘s κ = 0.72, 95% CI: 0.63–0.81), supporting the use of calf circumference as a valid supplementary indicator when CT was unavailable. To assess inter-rater reliability, two trained researchers independently performed CT muscle area delineation on a randomly selected subset of 50 patients (10% of the cohort). The intraclass correlation coefficients (ICCs) for quadriceps CSA and psoas CSA were 0.94 (95% CI: 0.89–0.97) and 0.92 (95% CI: 0.86–0.96), respectively, indicating excellent inter-rater agreement. For calf circumference measurements, the inter-rater ICC in the same subset was 0.88 (95% CI: 0.80–0.93). These results confirm that the muscle mass assessments were highly reproducible across raters.

*Aetiologic criteria:*

- Reduced nutrient intake/absorption: Documented decrease in oral food intake to ≤ 50% of estimated energy requirement for ≥ 1 week, or any reduction for ≥ 2 weeks, or presence of clinically diagnosed malabsorption syndromes (e.g., chronic diarrhea, pancreatic insufficiency). Data were extracted from admission nursing dietary intake records and physician progress notes.
- Inflammation/chronic inflammatory burden: Chronic inflammation was considered present if either of the following was met: (a) serum CRP ≥ 10 mg/L at admission (based on the widely accepted threshold for clinically significant systemic inflammation); or (b) a documented diagnosis of a chronic inflammatory condition that could affect nutritional status (e.g., active rheumatoid arthritis, inflammatory bowel disease, or chronic obstructive pulmonary disease with systemic involvement). For this cohort, the CRP threshold was the predominant operational criterion.

Given that low BMI was absent by inclusion criteria (all patients had BMI ≥ 28 kg/m^2^), the GLIM positivity in this cohort was driven primarily by combinations of involuntary weight loss, low muscle mass, reduced intake, and/or inflammation.

#### Concealed malnutrition and sarcopenic obesity metrics

(1) Concealed malnutrition: Concealed malnutrition was defined as a patient having a BMI still in the obese range ( ≥ 28 kg/m^2^) while simultaneously meeting the GLIM criteria for malnutrition, but without meeting the GLIM phenotypic criterion of significant involuntary weight loss (i.e., > 5% within the past 3 months or > 10% within the past 6 months). Patients who met the GLIM criteria and had significant recent involuntary weight loss were classified as having overt malnutrition, despite their BMI remaining in the obese range, because the weight-loss signal is clinically apparent rather than “concealed” by high BMI. This distinction was made to isolate the truly “BMI-masked” phenotype in which no conventional warning sign (low BMI or weight loss) is present, and malnutrition is only detectable through assessment of muscle mass and inflammatory burden.

(2) Low muscle mass indicator: Low muscle mass was assessed primarily by CT-measured quadriceps and psoas cross-sectional areas, using cut-offs recommended for Asian populations. For patients without complete CT data, calf circumference was used as a secondary assessment indicator. The study focused on the association between low muscle mass and nutritional risk in obese patients to evaluate the limitations of traditional BMI-oriented screening tools in the setting of muscle loss.

(3) Sarcopenic obesity: Sarcopenic obesity was defined as BMI ≥ 28 kg/m^2^ together with low muscle mass. The study further analyzed the ability of different nutritional screening tools to identify patients with sarcopenic obesity and specifically evaluated the potential missed diagnosis of nutritional risk in this population.

#### Inflammation and disease severity metrics

(1) CRP inflammatory burden indicator: CRP reflects systemic chronic inflammatory burden, with < 10 mg/L defined as low burden and ≥ 10 mg/L as high burden. Given that chronic low-grade inflammation drives obesity-related muscle wasting and nutritional abnormalities, we analyzed CRP differences across nutritional risk statuses and evaluated screening tools’ ability to identify inflammation-driven abnormalities.

(2) WOMAC functional score: WOMAC evaluates pain (0–20), stiffness (0–8), physical function (0–68), and total score (0–96); higher scores indicate worse impairment. We examined the relationship between nutritional risk and functional decline, focusing on the functional characteristics of patients with concealed malnutrition.

(3) Kellgren–Lawrence radiographic grade: K-L grade assesses KOA severity: grade 0 = no osteoarthritis, 1 = doubtful osteophyte, 2 = definite osteophyte with mild joint space narrowing, 3 = moderate narrowing with sclerosis, 4 = severe narrowing with marked deformity. We compared radiographic severity across nutritional risk categories to explore associations between nutritional abnormalities and structural joint degeneration.

#### Short-term perioperative clinical outcomes

(1) Length of hospital stay: Length of stay was defined as the total number of days from admission to discharge, reflecting perioperative recovery efficiency and hospital resource utilization. The study further examined length of stay differences among patients with different nutritional risk statuses.

(2) Perioperative complications: Perioperative complications included delayed wound healing, pulmonary infection, deep vein thrombosis of the lower extremity, and unplanned readmission within 30 days postoperatively. All events were confirmed from electronic medical records and follow-up records. The study focused on the occurrence of perioperative adverse events in patients with concealed malnutrition and sarcopenic obesity to evaluate the potential impact of unrecognized nutritional abnormalities on short-term clinical outcomes in the setting of obesity.

#### Statistical methods

All analyses used R (4.3.2) and SPSS (27.0), following STROBE guidelines. Normality was assessed by Shapiro–Wilk test. Normally distributed data are mean ± SD (*t*-test); non-normal data are median (IQR) (Mann–Whitney U). Categorical data are frequencies/percentages (χ^2^ or Fisher’s exact). Skewed variables were log-transformed. Missing data (<10%) were handled by multiple imputation (5 datasets). Outliers were screened by boxplots and Z-score. For pairwise comparisons of diagnostic metrics between screening tools (e.g., sensitivity, specificity, missed diagnosis rates, and detection rates), McNemar’s test for paired binary data was used, as all screening tools were applied to the same patients. When multiple pairwise comparisons were performed (e.g., each tool vs. GLIM, or tool vs. tool comparisons), *P*-values were adjusted using the Bonferroni correction to control the family-wise error rate. For the primary analyses comparing each of the five screening tools against the GLIM reference, the Bonferroni-adjusted significance level was set at α = 0.01 (0.05/5). For exploratory pairwise comparisons between individual screening tools, the adjusted significance level was α = 0.005 (0.05/10). Adjusted *P*-values < 0.05 were considered statistically significant after correction.

Cohen’s κ (pairwise) and Fleiss’ κ (multi-tool) assessed diagnostic agreement; weighted κ for ordinal variables. κ: < 0.20 poor, 0.21–0.40 fair, 0.41–0.60 moderate, 0.61–0.80 substantial, > 0.80 almost perfect. A discordance framework classified patients as agreed positive, agreed negative, or discordant. Missed diagnosis rates for concealed malnutrition were calculated, and differences in BMI, inflammation, muscle mass, and function across discordance patterns were compared.

Using GLIM as reference, ROC curves evaluated each tool’s performance (AUC, sensitivity, specificity, PPV, NPV). AUC: 0.50–0.70 low, 0.70–0.90 moderate, > 0.90 high. ROC curves were compared by DeLong’s test, and subgroup ROC analyses were performed in high-BMI, high-inflammation, and sarcopenic obesity groups. AUCs were reported with 95% confidence intervals (CIs) estimated using 2,000 bootstrap replicates with bias-corrected and accelerated (BCa) method. ROC curves were compared using DeLong’s test.

Net reclassification improvement (NRI) and integrated discrimination improvement (IDI) assessed the added value of muscle mass and CRP to a traditional BMI-based model (hereafter “base model”), which included age, sex, BMI, hypertension, and type 2 diabetes mellitus. The extended model added CRP (as a continuous variable and categorized at ≥ 10 mg/L) and low muscle mass (binary) to the base model.

To evaluate the incremental value of incorporating muscle mass and inflammatory burden into traditional screening, we constructed two logistic regression models for predicting GLIM-defined malnutrition: (1) Base model (traditional model): Included the following prespecified clinically available variables that are typically used in routine admission assessment: age (continuous, years), sex (binary), BMI (continuous, kg/m^2^), hypertension (binary), and type 2 diabetes mellitus (binary). These variables were selected a priori based on clinical plausibility and their routine availability in admission records, rather than through data-driven variable selection, to reflect a realistic “bedside” screening scenario. (2) Extended model: Included all base-model variables plus CRP (continuous, log-transformed to correct right-skewness) and low muscle mass (binary). CRP was selected to capture the GLIM aetiologic criterion of inflammation, and low muscle mass to capture the GLIM phenotypic criterion of reduced muscle reserve. (3) Model-building strategy: Both models were fitted using multivariable logistic regression with all prespecified variables entered simultaneously (forced entry), without stepwise selection, to avoid overfitting and to maintain clinical interpretability. Variable selection was hypothesis-driven rather than data-driven, based on the conceptual framework that inflammation and muscle loss are key drivers of concealed malnutrition in obesity. (4) Handling of collinearity: Multicollinearity was assessed using variance inflation factors (VIFs) for all predictors in both models. A VIF > 5 was considered indicative of significant collinearity. For variables with VIF > 5, we planned to examine correlation matrices and, if necessary, combine or exclude collinear variables. Low muscle mass and sarcopenic obesity were not entered simultaneously into the same model because sarcopenic obesity is defined using low muscle mass, and including both would introduce tautological collinearity (VIF > 5). Therefore, low muscle mass and sarcopenic obesity were evaluated in separate models. (5) Internal validation: Model performance was internally validated using 1,000 bootstrap resamples with replacement. For each bootstrap sample, the full model-building process (forced entry of prespecified variables) was repeated, and discrimination (AUC) and calibration (calibration slope and intercept) were estimated. Optimism-corrected AUCs and calibration statistics were calculated using the bootstrap bias-correction method. Model calibration was assessed by the Hosmer–Lemeshow goodness-of-fit test and visual inspection of calibration plots with loess smoothing. The Brier score was calculated as an overall measure of model performance. (6) Reclassification analysis: The added value of the extended model over the base model was quantified using the continuous NRI and integrated discrimination improvement (IDI), with 95% confidence intervals estimated from 1,000 bootstrap replicates.

To quantify the non-tautological contribution of these variables and to address potential circularity, we performed two sensitivity analyses: (a) restricting the model to the concealed malnutrition subgroup (*n* = 121), in which GLIM positivity was driven entirely by low muscle mass and/or inflammation because weight loss and low BMI were absent by definition; and (b) using a modified GLIM reference that excluded low muscle mass and inflammation from the diagnostic criteria (i.e., GLIM defined solely by weight loss plus reduced intake), to test whether the added variables still improved classification when the reference standard was partially orthogonal to the added predictors.

Univariate and multivariable logistic regression identified correlates of concealed malnutrition, sarcopenic obesity, and perioperative outcomes. Covariates included age, sex, BMI, diabetes, hypertension, and CRP. Results as OR (95% CI). Multicollinearity was checked (VIF > 5 significant).

Restricted cubic splines explored non-linear relationships between CRP, BMI, or muscle mass and concealed malnutrition risk. Internal validation used 1,000-bootstrap resampling; calibration assessed by calibration curve and Brier score. Sensitivity analyses (complete-case and repeated imputation) tested robustness. All tests were two-sided, *P* < 0.05 significant.

Missing data were handled using multiple imputation with 5 imputed datasets under the missing-at-random assumption. The proportions of missing data for each key variable were as follows: serum albumin 4.2% (22/520), total lymphocyte count 3.8% (20/520), total cholesterol 2.9% (15/520), CRP 3.1% (16/520), ESR 4.6% (24/520), BMI 0.8% (4/520), WOMAC score 5.8% (30/520), and CT-derived muscle mass 3.5% (18/520 for patients with CT available but incomplete CSA delineation; overall CT availability of 76.2% is described separately in the muscle mass assessment section). No variable exceeded the prespecified 10% missingness threshold. Imputation models included all variables used in the main analyses, and imputed values were pooled using Rubin‘s rules. Complete-case sensitivity analyses confirmed the robustness of the results, as reported in the sensitivity analysis section.

All 95% confidence intervals for κ values, AUCs, NRI, and IDI were estimated using 2,000 bootstrap replicates with the bias-corrected and accelerated (BCa) method.

## Data Availability

The raw data supporting the conclusions of this article will be made available by the authors, without undue reservation.
