# Supplementary material for: Diagnostic agreement of nutritional screening tools in obesity-related knee osteoarthritis
Source: Front Nutr. 2026 Sep 23;13:1887413. doi: 10.3389/fnut.2026.1887413 (PMC13643906; doi:10.3389/fnut.2026.1887413)
Supplement: Supplementary file 1 [file Table_1.docx]

**Supplementary Table S1. Scoring ranges, cut-off values, and classification criteria for the five nutritional screening tools**

| **Screening tool** | **Components** | **Scoring range** | **Risk cut‑off** | **Risk categories** |
| --- | --- | --- | --- | --- |
| **NRS‑2002** | Nutritional status (weight loss, food intake, BMI) + Disease severity + Age adjustment | 0–7 | ≥3 = at risk | <3: no risk; ≥3: at risk |
| **MUST** | BMI + Involuntary weight loss + Acute disease effect | 0–6 | ≥1 = medium/high risk | 0: low risk; 1: medium risk; ≥2: high risk |
| **MNA‑SF** | Appetite change, mobility, weight loss, neuropsychological status, BMI | 0–14 | ≤11 = at risk or malnourished | 12–14: normal; 8–11: at risk; 0–7: malnourished |
| **GNRI** | Serum albumin + Current/ideal body weight ratio | — | ≤98 = any risk | >98: no risk; 92–98: mild; 82–91: moderate; <82: severe |
| **CONUT** | Serum albumin + Total lymphocyte count + Total cholesterol | 0–12 | ≥2 = any abnormality | 0–1: normal; 2–4: mild; 5–8: moderate; 9–12: severe |

**Supplementary Table S2. 2 × 2 contingency tables for each screening tool against GLIM criteria**

| Screening tool | GLIM positive (n=146) | GLIM negative (n=374) | Total |
| --- | --- | --- | --- |
| NRS-2002 positive | 105 | 115 | 220 |
| NRS-2002 negative | 41 | 259 | 300 |
| Total | 146 | 374 | 520 |
|  |  |  |  |
| Screening tool | GLIM positive (n=146) | GLIM negative (n=374) | Total |
| --- | --- | --- | --- |
| MUST positive | 84 | 34 | 118 |
| MUST negative | 62 | 340 | 402 |
| Total | 146 | 374 | 520 |
|  |  |  |  |
| Screening tool | GLIM positive (n=146) | GLIM negative (n=374) | Total |
| --- | --- | --- | --- |
| MNA-SF positive | 111 | 102 | 213 |
| MNA-SF negative | 35 | 272 | 307 |
| Total | 146 | 374 | 520 |
|  |  |  |  |
| Screening tool | GLIM positive (n=146) | GLIM negative (n=374) | Total |
| --- | --- | --- | --- |
| GNRI positive | 123 | 87 | 210 |
| GNRI negative | 23 | 287 | 310 |
| Total | 146 | 374 | 520 |
|  |  |  |  |
| Screening tool | GLIM positive (n=146) | GLIM negative (n=374) | Total |
| --- | --- | --- | --- |
| CONUT positive | 127 | 97 | 224 |
| CONUT negative | 19 | 277 | 296 |
| Total | 146 | 374 | 520 |
